# Effect of aquatic-treadmill training on cerebrovascular function and gait in community-dwelling stroke survivors: a feasibility and preliminary efficacy study

**DOI:** 10.3389/fspor.2025.1680250

**Published:** 2026-01-13

**Authors:** Rachel L. Bevins, Karen Thomas, Claire V. Burley, T. David Punt, Samuel J. E. Lucas

**Affiliations:** 1 School of Sport, Exercise and Rehabilitation Sciences, University of Birmingham, Birmingham, United Kingdom; 2 School of Science, Coventry University, Coventry, United Kingdom; 3 Sport and Health, Birmingham Newman University, Birmingham, United Kingdom; 4 Centre for Human Brain Health, University of Birmingham, Birmingham, United Kingdom; 5 Dementia Centre of Excellence, Curtin enAble Institute, Curtin University, Perth, WA, Australia; 6 Centre for Movement and Wellbeing, University of Birmingham, Birmingham, United Kingdom

**Keywords:** aquatic treadmill, cerebral blood flow, exercise, gait, stroke rehabilitation

## Abstract

**Background:**

Water-based exercise augments exercise-induced increases in brain blood flow, optimizing a proposed key mechanistic pathway for improved brain health. Aquatic treadmill exercise has been shown to aid gait re-education of stroke survivors, however its potential to enhance cerebrovascular function in this clinical population has not been tested. This pilot study aimed to examine the feasibility and preliminary efficacy of a 4-week aquatic treadmill (ATM) training intervention on cerebrovascular responsiveness and gait function in stroke survivors.

**Methods:**

Six community-dwelling stroke survivors (58 ± 11 years, 8 ± 11 years post stroke) completed a 4-week ATM intervention, consisting of 20–30 min sessions, 3 times/week. Pre- and post-intervention measures were taken of cerebrovascular reactivity (CVR), indexed via changes in middle cerebral artery blood velocity (MCAv) to a hypercapnic (5% CO_2_ in air) stimulus. Changes in mobility were assessed via 10-metre walk, Timed-Up-And-Go, and 6-minute walk (6MW) tests.

**Results:**

Adherence to the intervention was excellent, with 70 of the 72 (97%) available training sessions completed by participants. CVR increased on average by 44% (95% CI: ±58%; 2.8%–4.0%ΔMCAv/mm Hg ΔPETCO_2_) in the stroke-affected hemisphere and 48% (95% CI: ±41%; 3.0%–4.5%ΔMCAv/mm Hg ΔPETCO_2_) in the unaffected hemisphere post intervention, although changes did not reach statistical significance (*p* = 0.218; Friedman's test). Within-group gait improvements were seen in speed and distance, with some changes above clinically meaningful thresholds; although this was not uniformly evident.

**Conclusion:**

This pilot study established ATM training as a feasible option for some patients in stroke rehabilitation. Despite the limited sample size, the study demonstrated promising enhancements in cerebrovascular function, with preliminary evidence suggesting concurrent improvements in gait performance. Well-designed, larger studies are warranted.

## 1 Introduction

Cerebral blood flow (CBF), and its responsiveness to changes in arterial carbon dioxide content [PCO_2_; the most potent regulator of CBF termed cerebrovascular CO_2_ reactivity (CVR)], decreases with ageing and is impaired in clinical conditions such as stroke (1–3). Moreover, impaired CVR has been found to predict ipsilateral stroke and transient ischemic attack risk (4, 5), as well as predict all-cause cardiovascular mortality (6). Thus, the regulatory capacity of the cerebrovasculature presents as a vital mediator of brain health and optimal function, and as a measurable outcome for interventions focused on improving brain health.

Controlling vascular risk factors and implementing lifestyle changes, particularly increased physical activity, are key strategies for preventing recurrent stroke events (7, 8). However, while it is well-established that regular physical activity improves physical function, fitness and quality of life after stroke (9–11), adherence to current guidelines [e.g., 30–40 min, 3–5 times/week at >70% heart rate peak (7)] is low (11). While low adherence rates are a common challenge for both the general population and clinical groups, stroke survivors face unique barriers to exercise, including physical impairments, fear of falling/stroke recurrence, and limited access or knowledge about how to exercise (12). Further, the reduced physical capacity may also constrain the physiological effectiveness of traditional exercise prescriptions. Therefore, alternative approaches are needed that optimize the stimulus-strain response to elicit meaningful adaptation (13).

The mechanical movement of blood through vessels (i.e., shear stress) is a key exercise-induced mechanism facilitating improved vascular function (14). Water-based exercise has been used to target this response for brain blood flow (15, 16), potentially optimizing this mediator of improved brain vascular health. Pugh and colleagues (17) first illustrated this using a low-to-moderate intensity, water-based, box-stepping task in young healthy adults, reporting higher cerebral blood velocity (CBv) during this water-based activity. Building on this, we used aquatic treadmill exercise to assess the CBv response across a range of exercise intensities (16), focusing on a mode (i.e., walking) that is transferrable for populations with impaired physical capacity. Notably, we showed that CBv increased during aquatic treadmill walking to a similar extent to that induced from dry-land running at the exercise intensity promoted by current public health guidelines (∼65% of aerobic capacity). While these initial studies show the potential for how water-based exercise could augment CBF during exercise, the effect of the repeated stimulus over weeks (i.e., training) on chronic cerebrovascular adaptation has received little attention—albeit with results to date in healthy older adults supporting this general concept (18). Given the link between CVR and stroke risk, aquatic treadmill exercise may be an effective exercise stimulus for vascular adaptation and improve brain vascular health.

Mobility impairments are common post-stroke, making gait a central focus of rehabilitation (19), However, functional gains often decline after formal rehabilitation ends, along with the protective benefits of physical activity. Aquatic treadmill (ATM) training offers controlled conditions and targets walking—a key functional activity—making it increasingly popular in stroke rehabilitation (12). Studies to date show ATM improves gait velocity, non-paretic step length, endurance, and aerobic capacity in subacute (3 weeks-to-6 months since onset) stroke survivors (20, 21), but its efficacy in chronic stroke populations (>6 months post-event) remains unclear.

Therefore, the primary aim of this study was to examine the feasibility and preliminary efficacy of ATM exercise to improve brain blood flow regulation in chronic stroke survivors following a 4-week training intervention. A secondary aim was to assess changes in gait function, building upon previous work showing ATM exercise improves gait post stroke (20). We hypothesized that ATM training would be feasible, well tolerated, and lead to improvements in cerebrovascular regulation and gait function in chronic stroke survivors.

## 2 Materials and methods

### 2.1 Study design

**Figure 1:**
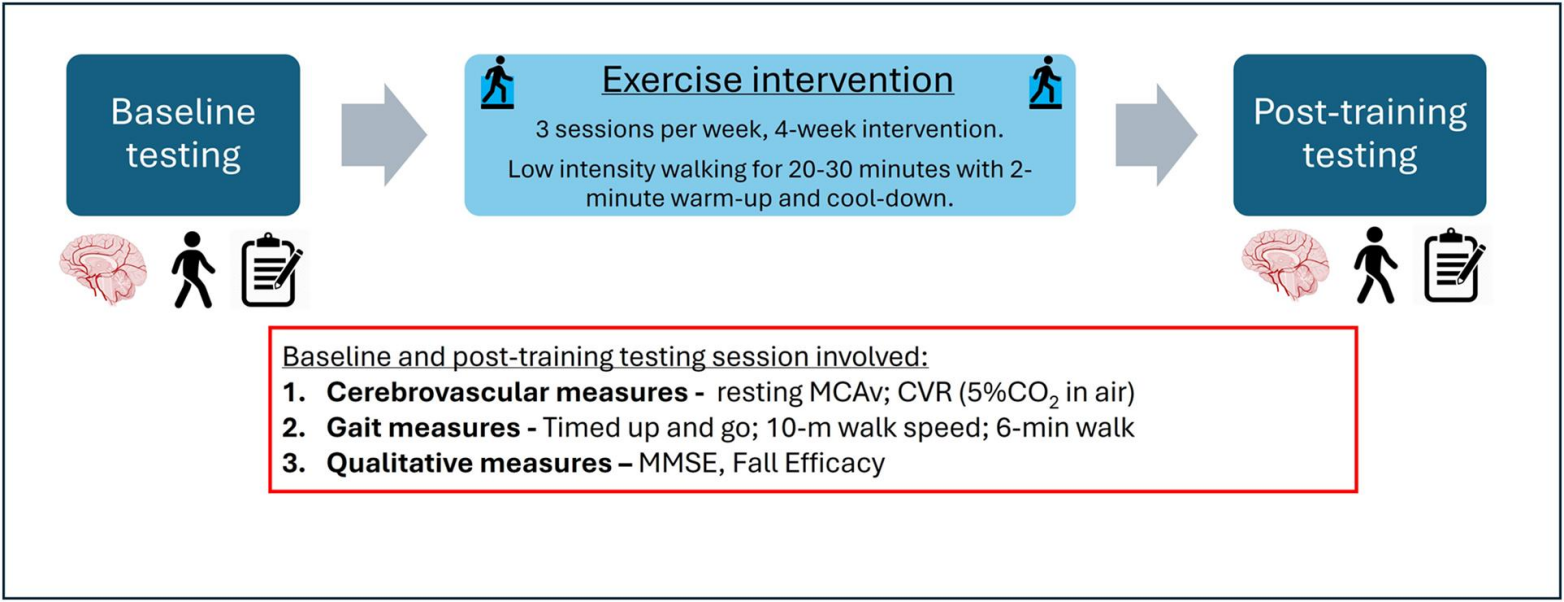
Schematic of the study design.

This study was a 4-week pre/post repeated measures, training intervention (Figure 1). Each participant used an aquatic treadmill (FOCUS, HYDRO PHYSIO™, UK) for a 20–30-minute session 3 times per week for 4 weeks, consistent with exercise training recommendations for this population (7, 12). Baseline (pre-) and post-intervention testing sessions consisted of a battery of standardized vascular and gait function tests, as well as questionnaires (see details below).

### 2.2 Participants

Six volunteer community-dwelling stroke survivors (4 males and 2 females) were recruited to this study (see Table 1 for demographic and clinical characteristics). Prospective participants were approached via telephone or email. To be included in the pilot study, stroke survivor volunteers had to be over 18 years of age, medically stable, able to provide informed consent, able to walk independently [i.e., Functional Ambulatory Category (FAC) of 4 and above], and be physically active [i.e., be at least classed as moderately active on the NHS General Practice Physical Activity Questionnaire (GPPAQ)]. Exclusion criteria included cognitive impairments preventing understanding of the task and mobility limitation attributable to a non-stroke pathology. Demographic information was collected at baseline testing, including age, date of stroke, current co-morbidities and cognitive competence utilizing the Mini Mental State Examination (MMSE). At the pre-training baseline testing, one participant was categorised as moderately active, while all others were active as scored by the GPPAQ. Participant testing was held in laboratories located at the University of Birmingham, whilst the ATM training program took place at a specialist physiotherapy clinic in Birmingham. Transport was provided for participants to the training and the testing sessions. Full ethical approval for this study was granted by the Science, Technology, Engineering and Mathematics Ethics Committee at the University of Birmingham (ERN_16-0020). Participants gave written, fully informed consent for this study, and all procedures were conducted in accordance with the principles laid down by the Declaration of Helsinki.

**Table 1:** Baseline demographic and clinical characteristics for each participant.

Participant ID	Age (years)	Sex	Weight (kg)	Height (cm)	Time since stroke (years)	Paretic side^a^	FAC	MSSE	FES-I
A	51	M	76.0	177.0	14	L	5	26	26
B	54	M	88.2	172.0	5	L	5	28	9
C	64	F	80.8	156.0	14	R	5	25	14
D	62	M	75.9	178.0	6	L	5	29	15
E	33	M	72.3	187.0	8	L	5	28	14
F	69	F	64.0	158.5	5	R	5	22	26
Mean ± SD	55 ± 13		76.2 ± 12.0	171.4 ± 12.0	8.7 ± 4.3			26.3 ± 2.6	17.3 ± 7.0

a Note that the affected cerebral hemisphere will be opposite to the paretic side.

### 2.3 Training intervention

Following baseline testing (described below in outcome measures), participants were asked to attend 3 ATM training sessions per week for 4 weeks. Each session began with resting measurements of heart rate and perceived exertion (22; 6–20 scale). Following this, a 2-minute warm-up was conducted on the ATM at a self-selected pace comfortable for the participant. Exercise continued at a low intensity (target RPE 11) chosen by the participant. Each training session was set at a minimum of 20 min and a maximum of 30 min, determined by participant feedback/tolerance. Participants were asked to rate their RPE at 5-minute intervals during training, and were advised they could stop at any time. Following each exercise training session, there was a 2-minute cool-down period at the participant's self-selected pace chosen for the warm-up. The water level for each training session was set to the individual's iliac crest height, and participants were provided with aqua booties to wear during all exercise sessions. Water temperature was set to ∼32°C for all exercise sessions. Immediately following each training session, participants were seated with post-training heart rate and RPE measurements taken after 5 min. Resting and exercising heart rate was measured by telemetry (Polar, Finland), via a chest strap worn by participants during all sessions. The post-intervention testing occurred within 7 days of each participant's final training session.

### 2.4 Outcome measures

The feasibility of the study was assessed by examining adherence, retention, and safety. The criteria for adherence was set at ≥70% based on previous studies using community-dwelling stroke survivors (23, 24). The criteria for retention utilized an attrition rate set at ≤15% based on the acceptable drop-out rate established by the PEDro scale (25). The intervention was considered safe if there were no adverse events associated with the ATM training. Every ATM session was supervised by the same therapist for consistency in reporting.

The primary efficacy outcome measure was cerebrovascular function. Baseline cerebral blood velocity (CBv) and cerebrovascular responsiveness to changes in the arterial content of carbon dioxide (PCO_2_; i.e., CVR) were used to assess changes in cerebrovascular function post intervention. The procedure was the same for pre- and post-intervention testing sessions. Specifically, upon arrival at the laboratory, participants lay supine for ∼20 min while being instrumented. Once instrumented, participants lay quietly for resting baseline measures (3 min duration) then breathed a mixture of 5% CO_2_ in air from a Douglas bag for 4 min, which was repeated following a 3-minute recovery period. Left and right middle cerebral artery blood velocity (MCAv) were measured via 2-MHz ultrasound probes placed bilaterally on the head (Dopplerbox, DWL, Compumedics LTD, Germany). Partial pressure of end-tidal CO_2_ (P_ET_CO_2_) and ventilation were measured via a fast-responding gas analyzer (ML206, ADInstruments, New Zealand) and heated pneumotachograph (3813 series, Hans Rudolph Inc., Kansas, USA), respectively, by sampling the expired air from a mouthpiece. Each participant completed two 5% CO_2_ challenges, with the second challenge used to quantify MCAv-CO_2_ reactivity (1st was a familiarization). Alongside these, beat-to-beat measures of blood pressure were recorded using photoplethysmography via a finger cuff placed on the middle finger of the left hand (Portapres, Finapres Medical Systems BV, The Netherlands), and heart rate derived from the beat-to-beat waveform of the MCAv signal. Throughout, real-time data were recorded in LabChart software (v7, ADInstruments) via an analogue-to-digital converter (Powerlab, ADInstruments).

Secondary efficacy measures included gait function and questionnaire data. Tests of gait function included the 10-metre walk test (10MWT), the Timed Up and Go Test (TUG), and the 6-minute walk test (6MWT). Within the test-battery, both 10MWT and TUG were conducted 3 times, whereas the 6MWT was conducted once. The 6MWT was conducted last to minimize fatigue effects on either the 10MWT or TUG. For the 10MWT, participants walked at a self-selected speed in a straight line, starting and ending the test 2 m either side of a GAITRite instrumented walkway (CIR system Inc., Franklin, New Jersey, USA) to account for acceleration and deceleration. Walking speed and step lengths were calculated from the 10MWT using the GAITrite system. A stopwatch was used for the TUG, with the timer starting when the participant lifted from sitting and stopped when the participant sat back down. For the 6MWT, participants walked at a self-selected speed with the aim of completing as many lengths of a 10-metre track as they could within the time, and total distance was recorded.

The Mini-Mental State Examination (MMSE) questionnaire was used to assess cognitive ability and the Fall Efficacy- International (FES-I) Scale assessed fear of falling.

### 2.5 Data analysis

Left and right side MCAv measures were classified into stroke-affected and non-affected hemispheres. Mean MCAv values were taken from the average of the last 60 s of the 3-minute resting period and for the 4-minute CO_2_ challenge. CVR was calculated as the slope of the percent change in MCAv from baseline (rest) per mm Hg change in end-tidal PCO_2_ induced by the 5% CO_2_-challenge.

Statistical analysis was performed using Jamovi (v 2.2; 26). Given the small sample size, all statistics were performed and are reported using non-parametric methods. Specifically, pre- and post-intervention cerebrovascular measures (i.e., resting MCAv and CVR) were assessed via a 2-way ANOVA (affected hemisphere × time) using Friedman's test. For all other measures, a Wilcoxon signed-rank test was used to assess changes between pre- and post-intervention measures.

Data are reported as mean ± SD, with a threshold for significance set *a priori* to *p* ≤ 0.05. However, given the exploratory nature of this study, confidence intervals and descriptive statistics [median, interquartile range (IQR)] are also presented where appropriate. Finally, data for gait speed in the 10MWT and distance for the 6MWT were compared to recommended meaningful change values (27), as was 10MWT data for step lengths (28).

## 3 Results

### 3.1 ATM training feasibility

No participants withdrew at any stage of the study, with all participants completing pre- and post-intervention testing. One participant (D) was unable to complete 2 training sessions (due to acute illness), so completed 10 of the 12 training sessions only. The 5 other participants completed all 12 training sessions. This indicated excellent adherence to training, with 70 of the 72 available training sessions completed (97%). The majority of training sessions attended reach the maximum of 30 min (66/70), with the remaining 4 completing at least 20 min. There were no adverse events reported. All participants (6/6) reported enjoyment and feeling motivated by the ATM training; however, increased feelings of fatigue by the training were also reported (most notably by participant F).

**Figure 2:**
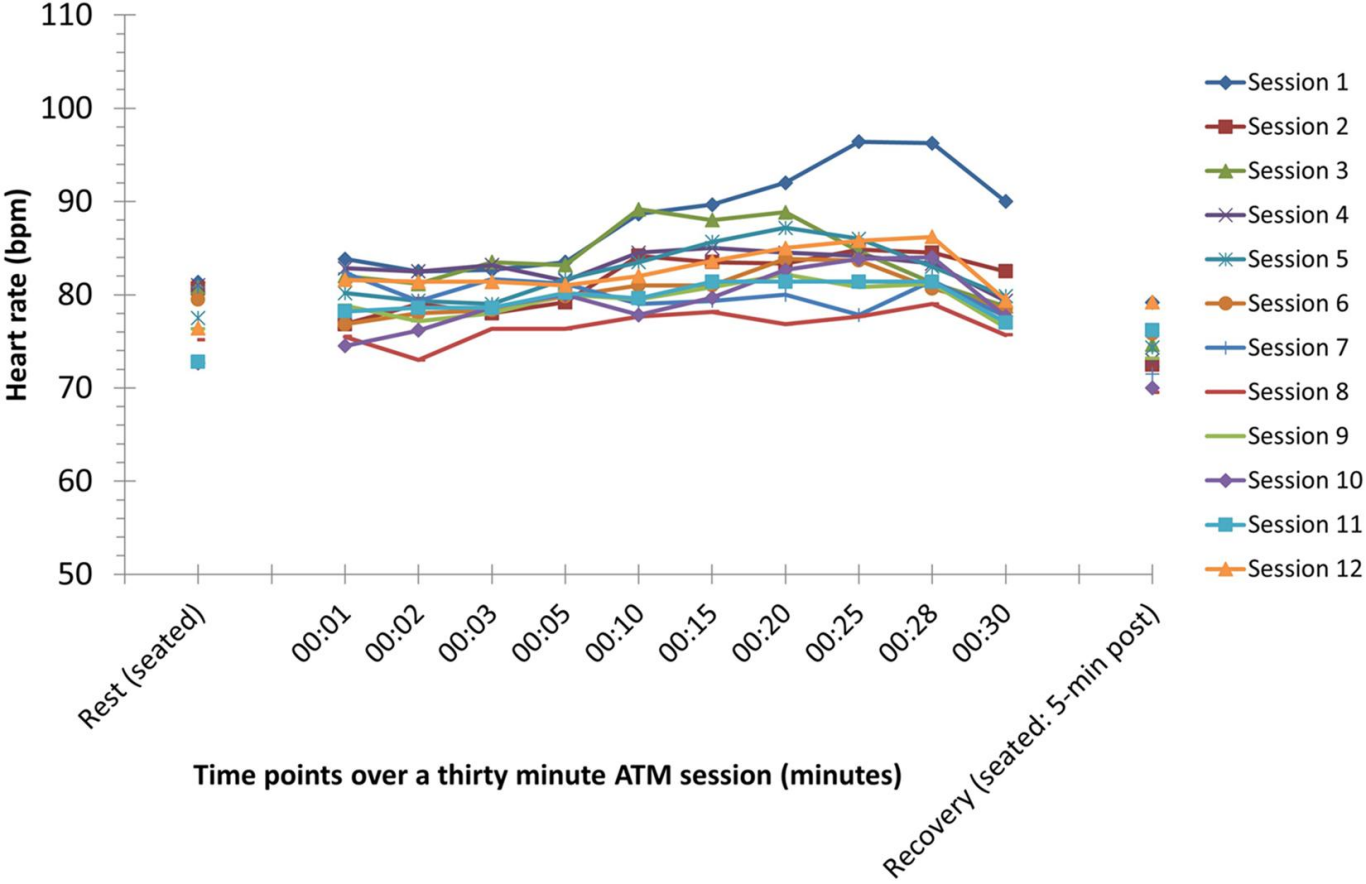
Mean heart rate response measures over a twelve session, 20–30-min aquatic treadmill training intervention in six community dwelling stroke survivors. Only mean data are reported for clarity.

Heart rates were measured for all 6 participants, at standardized intervals throughout every training session. Mean heart rate increased throughout the period of the ATM training session, peaking at approximately 20–25 min, prior to the cooldown period (Figure 2). Average heart rate in session 1 was higher than all other sessions, with later sessions showing lower resting heart rates and a lower average over the training duration (average peak heart rate ∼85 bpm). Individual session data are presented in the supplement.

### 3.2 Cerebrovascular function

Bilateral measures of MCAv were acquired from five participants. In the remaining participant (F), a reliable MCAv signal was not acquired throughout the protocol for both pre- and post-intervention testing. Thus, statistical analysis for resting and CVR data were conducted on five participants. Pre and post-intervention resting cerebro- and cardiorespiratory measures are presented in Table 2.

**Table 2:** Cerebrovascular and cardiorespiratory measures collected during resting baseline at pre- and post-intervention testing sessions.

Participant ID	MCAv_mean_ (stroke-affected hemisphere) (cm/s)	MCAv_mean_ (Non-affected hemisphere) (cm/s)	MAP (mm Hg)	Heart rate (bpm)	Ventilation (L/min)	P_ET_CO_2_ (mm Hg)
Pre-intervention						
A	47.0	68.1	89.4	59	5.4	41.6
B	26.7	27.8	95.1	61	12.4	37.0
C	52.9	40.1	75.2	75	9.8	39.5
D	40.5	34.0	97.7	55	12.8	37.6
E	65.8	63.7	69.5	60	8.4	41.7
Mean ± SD	46.6 ± 14.5	46.8 ± 18.1	85.4 ± 12.5	62 ± 8	9.8 ± 3.0	39.5 ± 2.2
Median (IQR)	47.0 (33.6–59.4)	40.1 (30.9–65.9)	89.4 (72.4–96.4)	60 (57–68)	9.8 (6.9–12.6)	39.5 (37.3–41.7)
Post-intervention						
A	44.3	59.8	83.9	58	7.3	37.4
B	27.1	30.3	67.6	59	13.5	39.3
C	48.6	52.7	73.0	65	10.0	37.6
D	42.3	54.0	87.7	58	12.0	38.7
E	65.3	65.9	68.2	59	10.0	43.9
Mean ± SD	45.5 ± 13.7	52.6 ± 13.5	76.1 ± 9.2	60 ± 3	10.6 ± 2.3	39.4 ± 2.6
Median (IQR)	44.3 (34.7–57.0)	54.0 (41.5–62.9)	73.0 (67.9–85.8)	59 (58–62)	10.0 (8.7–12.8)	38.7 (37.5–41.6)

Note that participant F is not included in these data as did not have a bilateral MCAv measure for either pre or post-intervention time points.

Resting MCAv_mean_ was not significantly different for either the stroke affected and non-affected hemisphere before and after the training intervention (*p* = 0.160). Resting MAP was on average lower post intervention, although did not reach statistical significance (*p* = 0.063).

**Figure 3:**
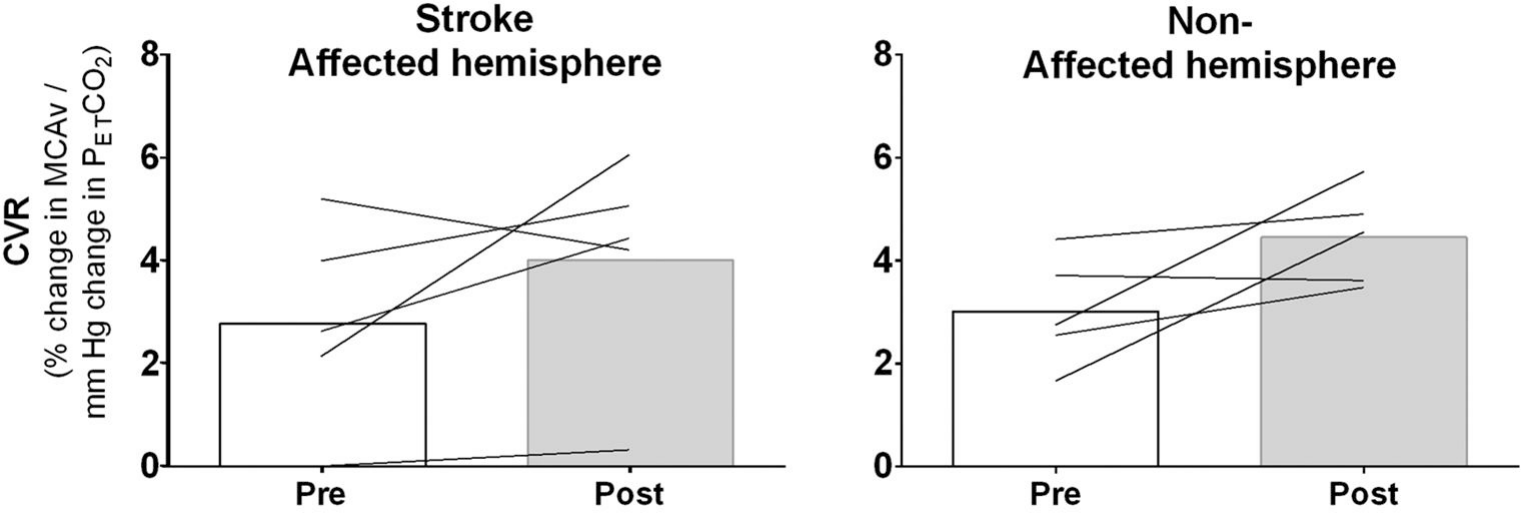
Mean (column) and individual (lines) measures of cerebrovascular CO_2_ reactivity (CVR) for stroke-affected (left panel) and non-affected (right panel) cerebral hemispheres before (pre) and following (post) a 4-week aquatic treadmill training intervention in community dwelling stroke survivors. Data presented here are from the five participants that had bilateral measures of middle cerebral artery velocity (MCAv) for both pre and post testing sessions.

As shown in Figure 3, four of the five participants increased MCAv-CO_2_ reactivity (i.e., CVR) for both the stroke affected and non-affected hemispheres following the intervention [on average up 44% (95% CI: ±58%) and 48% (95% CI: ±41%), respectively]. Nevertheless, these changes were not significant (*p* = 0.218; Friedman's test).

### 3.3 Gait function

**Figure 4:**
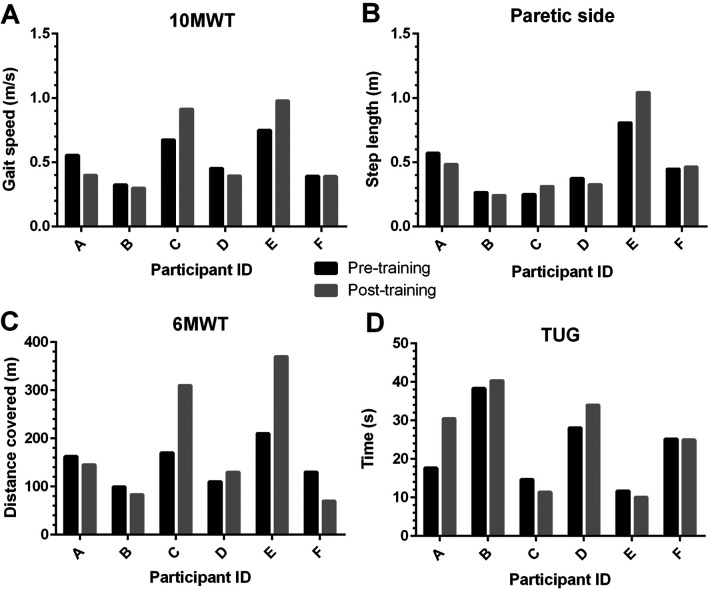
Individual data for 6 community-dwelling stroke survivors completing a battery of mobility tests to assess gait function before and following a 4-week aquatic treadmill training intervention. Mobility tests were the 10-metre walk (10MWT, panel **A**) and 6-minute walk (6MWT, panel **C**) tests, and the Timed-Up-And-Go (TUG, panel **D**) test. Walking speed and step lengths (panel **B**) were calculated from the 10MWT using a GAITrite instrumented walkway system.

Gait speed in the 10MWT increased from pre- to post-training (0.52 ± 0.17 vs. 0.56 ± 0.30 m.s^−1^; median of differences: −0.01 m.s^−1^; IQR: −0.08 to 0.23 m.s^−1^). This gait speed increase for the sample did not quite meet the criteria of 0.06 m.s^−1^ for a meaningful change (27). However, as shown in Figure 4A, two participants (C and E) increased their gait speed by over 0.20 m.s^−1^, which represents a substantial meaningful change for this metric. Paretic step length increased with training (pre: 0.45 ± 0.21 m; post: 0.48 ± 0.29 m; median of differences: 0.003 m; IQR: −0.056 to 0.106 m). Consistent with gait speed observations, participants C and E showed meaningful changes (28), with their paretic step length increasing more than a minimal detectable change (>6.75 cm; see Figure 4B).

Distance covered in the 6MWT increased from 146.9 ± 41.7 to 184.9 ± 124.8 m across the training intervention for the group as a whole (median of differences: 2.24 m; IQR: −27.75 to 145.0 m; see Figure 4C). The mean distance increase of 38 m after training is more than the 20 m increase that represents a small meaningful change for this metric (27). As shown in Figure 4C, 3 participants (C, D, and E) achieved this 20 m increase, with C (140 m increase) and E (160 m increase) well in excess of a substantial meaningful change (≥50 m). The three other participants walked shorter distance post-training, but only one (F) was clinically meaningful.

Finally, the average time taken to complete the TUG test increased following the intervention (from 22.6 ± 9.9 to 25.3 ± 12.3 s; median of differences: 0.91 s; IQR: −2.03 to 7.70 s). Similar to the other gait outcomes, there was variation between individuals—with only one participant (A) clearly showing a clinically meaningful change (i.e., >23% change; 29) with this gait test (Figure 4D).

## 4 Discussion

### 4.1 Main findings

This study aimed to examine the feasibility and preliminary efficacy of a 4-week aquatic treadmill (ATM) training intervention on cerebrovascular responsiveness and gait function in stroke survivors. All six community-dwelling stroke survivors recruited for this study completed the pre- and post-training testing, and only two training sessions were missed by a single participant (i.e., 70 of the 72 available training sessions were completed). The primary efficacy outcome measure of CVR increased by 44% in the stroke-affected hemisphere and 48% in the non-affected hemisphere, albeit not reaching statistical significance in this small cohort. Within-group gait improvements were seen in speed and distance, although these were not uniformly evident. Collectively, this study highlights the feasibility and potential efficacy for this water-based, vascular-targeted intervention to induce impressive improvements in cerebrovascular function that are linked with brain function and stroke mortality risk. These data also provide valuable information (e.g., sample size estimation) to inform subsequent trials looking to evaluate the full efficacy of ATM for improving brain vascular health.

### 4.2 Feasibility of ATM training

The understanding that regular exercise improves health outcomes in stroke survivors is well-established (12), yet adherence to the currently promoted exercise recommendations in this population is poor (11). Therefore, improving the accessibility and potency of the exercise stimulus for improved health and functional outcomes by utilizing water-based activity is one potential solution to this complex problem. Encouragingly, our study extends previous feasibility findings of ATM training in sub-acute (<3 months since onset) (21) to chronic stroke survivors. Over the 4-week training period utilized for this study, there were no participant withdrawals, and the training session attendance was 97%. While this is a relatively short training period, these initial observations show clear potential for the feasibility and efficacy of this type of training in this cohort and therefore warrant further investigation over a longer intervention to determine longer-term adherence and compliance. In addition, the range of heart rate responses we observed (see Supplementary Figure S1) reflected our primary focus on the feasibility of this cohort to tolerate and adhere to ATM training. Further research is needed to understand what the optimal dose and/or intensity is for this type of exercise to deliver meaningful and sustained changes in function.

### 4.3 Impact of ATM training on cerebrovascular function

Water-based exercise has a number of physiological advantages to support exercise-induced adaptation. Specifically for brain vascular-related adaptation, the centralization of blood volume improves cardiac output (30, 31), one key regulatory of CBF (32), and therefore augments blood flow through the cerebral vessels. We (16) and others (15, 17) have demonstrated this in an acute context, with the latter studies demonstrating that the increases in MCAv may also be linked with increased retention of PCO_2_ in the arterial circulation—another key regulator of CBF (32). Regardless of the mechanism(s) that results in higher cerebral perfusion, based on findings from the peripheral vasculature (33), this higher CBF will enhance the shear stress stimulus linked to positive exercise-induced vascular adaptation (14). Importantly for a population such as stroke survivors that may have impaired physical capacity as a result of their stroke, this augmented flow can be achieved at lower exercise intensities (16)—thereby improving the accessibility of the optimal exercise-induced adaptations. Indeed, compared to a 6-month traditional exercise training study in stroke survivors where this same CVR outcome measure was assessed (34), the gains we observed were far greater (∼27%–28% vs. 44%–48% increase in MCAv-CO_2_ reactivity). Furthermore, the magnitude of the changes in CVR observed here following ATM training (1.2%–1.4%ΔMCAv/mmHg Δ P_ET_CO_2_; Figure 3) are greater than: (1) the baseline variability (0.9%ΔMCAv/mmHg ΔP_ET_CO_2_), and (2) the exercise training-induced changes (0.76%ΔMCAv/mmHg ΔP_ET_CO_2_) we have previously observed in healthy young and older adults (35). Thus, while the present pilot study is a small cohort, the findings highlight the potential of more targeted exercise approaches to improve vascular health, and particularly for CVR that has established links to brain function (36, 37) and stroke risk (4, 5).

### 4.4 ATM training and gait function

The secondary aim of this study was to assess changes in gait function over this short intervention period. The improvements seen in some gait measure scores are promising with regard to the potential related benefits of the intervention for functional rehabilitation. Increases in walking endurance capacity were clinically meaningful. Based on distance completed in the 6MWT, one participant improved from a functional classification of limited community ambulator to unlimited community ambulator, with another participant improving from home ambulator to unlimited community ambulator (38). As stroke survivors place a high priority on the ability to regain walking independence, as well as the high health and social burden that more limited walking ability presents (39), these improvements are encouraging. While it was disappointing that not all participants demonstrated an improvement in walking post-intervention (see Figure 4), this was a relatively short duration training intervention. Nevertheless, we observed impressive changes in participants that were 14- and 8-years post stroke (participant C and E, respectively), highlighting that despite the chronic status of their condition, just 4-weeks of ATM training had clinically meaningful impacts on their gait function. Nevertheless, we acknowledge that recovery of walking post-stroke will be influenced by many factors (38), and this pilot study was not designed to address the potential range of responses to ATM training.

In contrast to the walking tests, we observe slower (impaired) times in the TUG after the intervention. The TUG incorporates many aspects of functional mobility including standing up from a chair, turning, gait initiation, walking and sitting back down (40), and is dependent on lower limb muscle strength, particularly the paretic limb (41, 42). Previous research has indicated that increases in walking speed in the 10MWT are detected earlier than increases in TUG time with training interventions (24), so it is possible that this short training intervention was not sufficiently long enough to improve TUG times. Further, given the increased resistance associated with water-based exercise, paretic limb strength gains would be anticipated, but may take longer than 4 weeks (i.e., 12 sessions) to develop. Future work could examine these potential strength gains independently, via more specific testing of limb strength. This additional benefit of water-based training provides an additional incentive for promoting this exercise modality, however, we emphasize that it should not replace targeted strength training sessions recommended for this population [e.g., 2–3 days per week of at least 1 set of 8–10 different exercises (12)]. Nevertheless, ATM could be a useful adjunct to these recommended guidelines for muscle-strengthening activity in stroke survivors.

### 4.5 Considerations and future directions

This was a short-duration exercise training study, so it is acknowledged that the observed high adherence rates to the training sessions could be a result of the lower commitment needed (compared to a 6-month or 1-year training intervention). Nevertheless, the findings of this pilot study do provide compelling feasibility data to take the next step in the research translational pipeline to demonstrate how mechanistically focused, basic science concepts can be implemented into a rehabilitation strategy (43). Notwithstanding the aforementioned limitations and the small sample size of this feasibility study, in just 4 weeks of training, we observed some impressive changes in physiology and gait function that may have real world impact in the lives of stroke survivors. Confirming such positive functional effects as well as determining adherence and the potential durability of the functional changes over a longer duration requires further study via a full randomized control trial, inclusive of follow-up once the exercise intervention support ends.

The nature of this study meant we were primarily interested in the feasibility of ATM *per se*, which meant we targeted stroke survivors that had high FAC ratings. It is acknowledged that recruiting participants with high FAC scores skews the sample toward individuals with greater baseline mobility, who may respond more favorably to rehabilitation. Therefore, future studies could consider inclusion of participants with more severe physical impairments, as the supportive nature of a water environment could provide an increased benefit both physically and psychologically for those unable to walk 10 m unassisted (44). However, those with lower baseline mobility may require adjustments to the rehabilitation protocol and the duration of the intervention to achieve beneficial outcomes.

Finally, we examined the efficacy of ATM training to improve cerebrovascular health via the transcranial doppler-derived measure of CVR. While this outcome measure has been identified as a key marker of brain health and linked with brain function and stroke risk, it only reflects one aspect of brain health that might benefit from a water-based rehabilitation strategy. Further work should utilize a multimodal brain imaging approach [i.e., Doppler, MRI and/or NIRS; see (45)] to comprehensively assess how ATM training improves brain vascular structure and function in stroke survivors.

### 4.6 Conclusion

Aquatic treadmill exercise has previously been shown to aid gait re-education of stroke survivors, however its potential to enhance cerebrovascular function in this clinical population is unknown. We utilized a water-based strategy to target a key mechanistic pathway for vascular adaptation, showing some promising potential for improvements in cerebrovascular responsiveness after just 4 weeks of training. While not uniformly evident, we also saw clinically meaningful changes in gait function for some participants across this short-duration intervention. Overall, this study has shown that ATM training can be an acceptable intervention for chronic stroke survivors and provides preliminary efficacy that it may positively impact brain health and gait function.

## Data Availability

The raw data supporting the conclusions of this article will be made available by the authors, without undue reservation.

## Supplementary material

Supplementary Figure S1 Individual heart rate responses for each participant for each of the twelve training sessions completed.
