# Supplementary figures and images for: Effect of aquatic-treadmill training on cerebrovascular function and gait in community-dwelling stroke survivors: a feasibility and preliminary efficacy study
Source: Front Sports Act Living. 2026 Jan 13;7:1680250. doi: 10.3389/fspor.2025.1680250 (PMC12835293; doi:10.3389/fspor.2025.1680250)

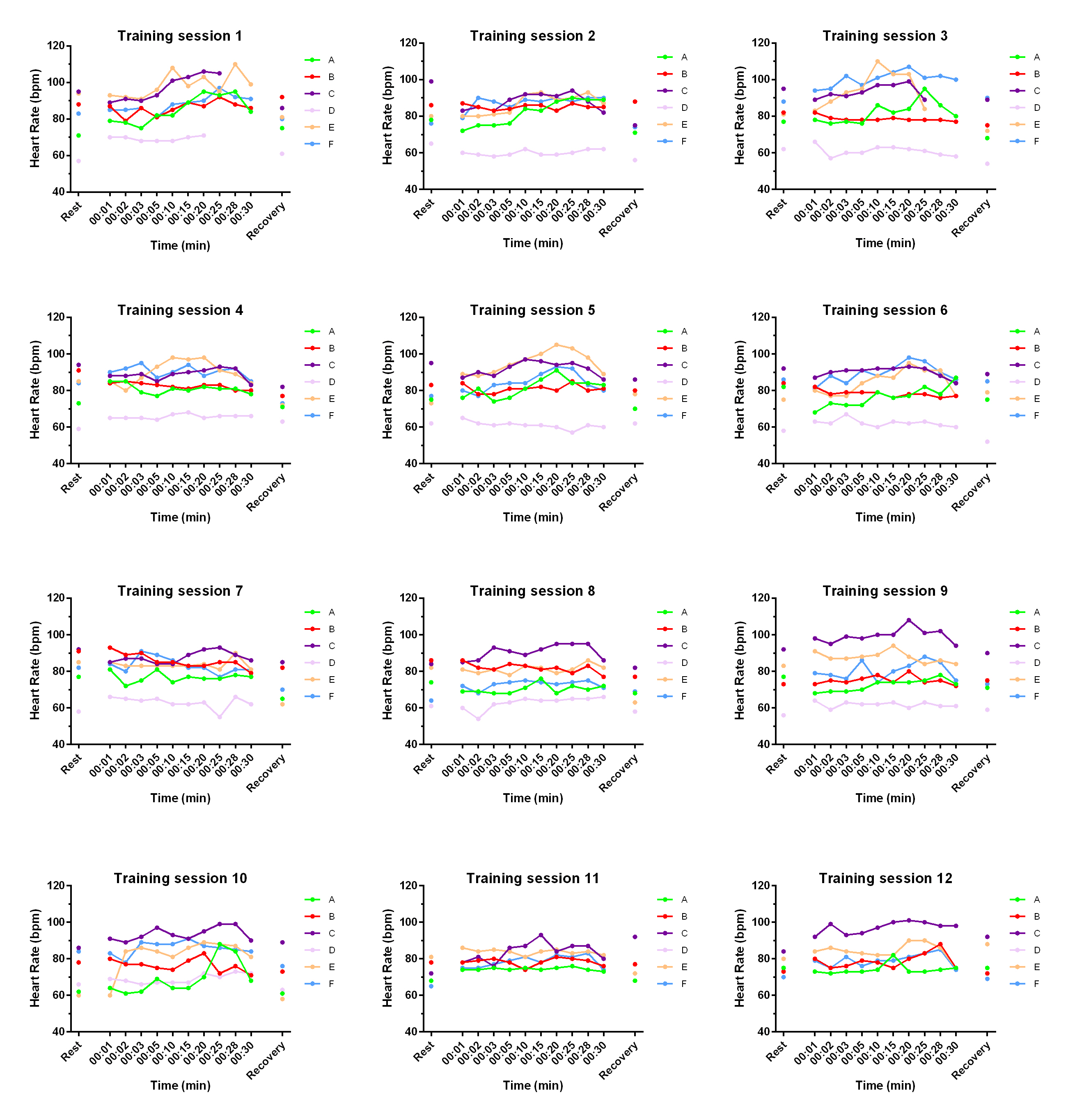

Supplement: Supplementary Figure S1 — Individual heart rate responses for each participant for each of the twelve training sessions completed. [file Image1.jpeg]
